# Copy Number Alterations as Novel Biomarkers and Therapeutic Targets in Colorectal Cancer

**DOI:** 10.3390/cancers14092223

**Published:** 2022-04-29

**Authors:** Elaine S. Tan, Todd C. Knepper, Xuefeng Wang, Jennifer B. Permuth, Liang Wang, Jason B. Fleming, Hao Xie

**Affiliations:** 1 Department of Gastrointestinal Oncology, H. Lee Moffitt Cancer Center and Research Institute, 12902 USF Magnolia Drive Tampa, Tampa, FL 33612, USA; elaine.tan@moffitt.org (E.S.T.); jenny.permuth@moffitt.org (J.B.P.); jason.fleming@moffitt.org (J.B.F.); 2 Department of Individualized Cancer Management, H. Lee Moffitt Cancer Center and Research Institute, 12902 USF Magnolia Drive Tampa, Tampa, FL 33612, USA; todd.knepper@moffitt.org; 3 Department of Biostatistics and Bioinformatics, H. Lee Moffitt Cancer Center and Research Institute, 12902 USF Magnolia Drive Tampa, Tampa, FL 33612, USA; xuefeng.wang@moffitt.org; 4 Department of Tumor Biology, H. Lee Moffitt Cancer Center and Research Institute, 12901 USF Magnolia Drive Tampa, Tampa, FL 33612, USA; liang.wang@moffitt.org

**Keywords:** copy number alteration, colorectal cancer, biomarkers

## Abstract

**Simple Summary:**

Copy number alterations (CNAs) occur due to changes to DNA structure that lead to the gain/amplification or loss/deletion of copies of DNA sections from a normal genome. These CNAs have been shown to impact gene expression and appear to play a significant role in the pathogenesis of colorectal cancer; additionally, certain CNAs appear to influence survival as well as response to certain treatments in colorectal cancer. As our understanding of CNAs in colorectal cancer increases, therapeutic options may be developed and implemented to target these CNAs and hopefully improve patient outcomes. The aim of this review is to describe the current methods to detect CNAs and summarize the current literature regarding CNAs and their roles from pathogenesis to prognosis and prediction in colorectal cancer.

**Abstract:**

In colorectal cancer, somatic mutations have played an important role as prognostic and predictive biomarkers, with some also functioning as therapeutic targets. Another genetic aberration that has shown significance in colorectal cancer is copy number alterations (CNAs). CNAs occur when a change to the DNA structure propagates gain/amplification or loss/deletion in sections of DNA, which can often lead to changes in protein expression. Multiple techniques have been developed to detect CNAs, including comparative genomic hybridization with microarray, low pass whole genome sequencing, and digital droplet PCR. In this review, we summarize key findings in the literature regarding the role of CNAs in the pathogenesis of colorectal cancer, from adenoma to carcinoma to distant metastasis, and discuss the roles of CNAs as prognostic and predictive biomarkers in colorectal cancer.

## 1. Introduction

Colorectal cancer is one of the most common and deadly cancers with over 1.8 million newly diagnosed cases worldwide per year and over 900,000 deaths worldwide in 2020 [1]. Over the last couple of decades, our understanding of colorectal cancer has improved as we have learned more about the genetic basis in colorectal cancer pathogenesis.

Somatic mutations have been shown to play a key role as prognostic biomarkers as well as therapeutic targets in colorectal cancer. Increasing evidence has shown that CNAs are also prevalent in colorectal cancer and can also have implications in the pathogenesis, prognosis, and therapeutic options. Amplification or gains of these CNAs can lead to increased expression of their respective genes while deletion or loss of CNAs can lead to decreased expression of their respective genes.

CNAs have been postulated to form through a variety of mechanisms related to somatic changes in DNA structure. Replication stress has been shown to lead to accumulation of double strand breaks at common fragile sites, which can further potentiate the formation of amplifications and deletions in these regions [2].

In vitro studies have shown that short and long CNAs appear to have different mechanisms of formation. Short CNAs less than 6 base pairs are thought to form by processes such as non-homologous end joining or template switching [3]. Some long CNAs have been shown to develop from chromosome breakage during mitosis, where the DNA fragments become missegregated and incorporated into micronuclei [4]. This may lead to chromothripis, causing further DNA replication errors that eventually become incorporated into the main nucleus, leading to thousands of chromosomal changes and generation of CNAs in a single event [5].

Replication timing also appears to influence CNA formation. Early replication timing has been associated with CNAs formed through homologous recombination, where unmatched DNA regions can be mistakenly combined and lead to duplications such as trisomy/tetrasomy or high-level amplifications or deletions in up to several hundred copies [6,7] and is related to early replication. Late replication timing is associated with CNAs formed through nonhomologous recombination as well as long CNAs greater than 20 Mb [4,8]. Lastly, genes involved in chromatin remodeling may also be involved in the pathogenesis of CNAs. Overexpression of *KDM4A*, a chromatin modulator, has been shown to influence specific copy number gains in malignancies [9].

In this review, we summarize methods to determine the presence and quantity of CNAs and summarize key findings in the literature on the significance of CNAs as disease drivers in colorectal cancer. We also discuss the roles of CNAs as prognostic and predictive biomarkers in colorectal cancer.

## 2. Methods to Determine CNAs

**Figure 1:**
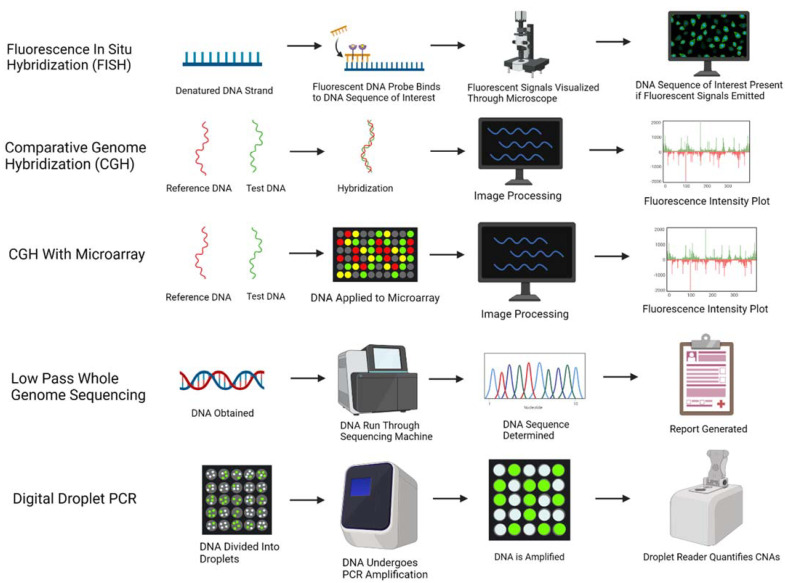
Various methods to characterize and quantify copy number alterations are illustrated: fluorescence in situ hybridization (FISH), comparative genome hybridization (CGH), CGH with microarray, low pass whole genome sequencing, and digital droplet PCR. Created with BioRender.com.

Five methods to determine CNAs are briefly described in Figure 1. Fluorescence in situ hybridization, or FISH, was first developed in the 1980s, where a fluorescent dye is attached to a DNA probe, which undergoes incubation with the DNA sample. If the target of interest is present in the DNA sample, the DNA probe will bind to the target and emit a fluorescent signal, which can then be visualized through a microscope [10]. FISH tends to have detection at a lower resolution of 5–10 Mb and is limited to analysis of specific DNA targets, as opposed to an entire genome; however, it can be performed more rapidly than other methods and does not require use of cell culturing as it can be performed on interphase nuclei [11].

Comparative genomic hybridization (CGH) was developed in the early 1990s and has been implemented in the identification of CNAs. CGH involves labeling a control genome (usually red) and a test genome (usually green) and then hybridizing them to metaphase chromosomes. The labeled DNA emits a fluorescent signal intensity: if both genomes are equal, a yellow fluorescence signal is emitted. If an amplification is present, more of the test DNA binds to itself leading to emission of a red signal; if a deletion is present, less of the test DNA binds to itself, leading to emission of a green signal. A fluorescence intensity plot is then generated and allows for identification of the copy number changes [12]. This technique is able to evaluate an entire genome and does not require cells that are actively dividing. However, CGH is limited in that it is unable to identify alterations outside of 5–10 megabases [12].

A newer method was developed a few years later implementing the use of microarrays with CGH. Microarrays are created with small amounts of DNA segments in an ordered fashion, with probes varying greatly in size from 25–85 base pairs to 200,000 base pairs, providing a significant advantage over traditional CGH [13]. Similar to CGH, the control and test DNA are labeled with fluorescent dye. The DNA samples are denatured, mixed together, and then added to the microarray. The DNA hybridizes with the probes in the array and emits various fluorescent signals based on amplification, gain, loss, or deletion of copy numbers; this leads to generation of a fluorescence intensity plot that subsequently identifies the CNAs present in the test DNA [13].

Low pass or low coverage whole genome sequencing has been another method used in the identification of CNAs. While CGH microarrays require specific probes, whole genome sequencing is able to map out CNAs across an entire DNA sequence. Low coverage whole genome sequencing has 0.5× coverage combined with a computational method called imputation to determine a DNA sequence, at a low cost and without compromising accuracy [14]. This is in contrast to high coverage whole genome sequencing which has 30× coverage, but at a greater cost [15]. While CGH has been considered the gold standard for identifying CNAs, low pass genome sequencing has also been validated in its ability to identify CNAs accurately and may be used more frequently in the future [14].

Another method that has been implemented to identify CNAs of specific genes is droplet digital PCR. With this method, a sample of DNA is divided into thousand to millions of droplets in water-in-oil partitions, where some droplets may have zero or one or more copies of the target of interest [16]. The DNA samples in the partitions then undergo simultaneous PCR amplification; based on the percentage of partitions that emit a fluorescent signal, a droplet reader is able to determine the presence and quantity of CNAs [17,18]. The random distribution of the DNA fragments allows for determination of the absolute concentration of the target DNA and confidence interval through a Poisson distribution, thus negating the need for standard curves [17]. This method also appears to have high precision and accuracy: for example, nearly similar concordance to FISH (Cohen’s kappa coefficient 0.76, 95% CI: 0.5–1.0) was seen with assessment of *MET* amplification in colorectal cancer [18].

Despite the multiple advances made in methods for detection of CNAs, there are some technical limitations related to these methods and also interpreting the results. Manual macrodissection is often implemented by the pathologist in preparing the tumor sample by viewing an H&E-stained slide with a microscope and then marking the appropriate areas for dissection. This is followed by a manual dissection of the areas without use of the microscope, which may lead to inadequate sampling results. Laser capture microdissection, on the other hand, is a more efficient method of dissecting a specimen with use of digital slide marking and has been shown to be more accurate in determining levels of protein expression [19]. However, implementation of this method can be costly and may not be readily available [19].

Additionally, if the lack of CNAs is reported, it is difficult to confirm without doing multiple biopsies if this is because the CNAs are truly not present or if they are present only in certain locations of the tumor that were missed due to sampling location and tumor heterogeneity [20]. Additionally, as cancer cells are exposed to treatment, various resistant subclones with a distinct genetic profile may emerge; however, when obtaining a single biopsy, it can be difficult to determine whether the sample contains the subclones, which would influence the CNA results [20]. Therefore, CNA results should be interpreted with these considerations in mind.

## 3. Pathogenesis

Multiple CNAs play a role in the development of colorectal adenocarcinoma, with certain CNAs being more prominent in early carcinogenesis and others being more prominent during disease progression and metastasis (Table 1).

### 3.1. Adenoma to Carcinoma

Gains of 8q and 20q and loss of 8p and 17p have been shown to play a role in the transition of adenomas to carcinomas. C-MYC is a primary proto-oncogene expressed on chromosome 8q24.21 and facilitates cell proliferation and survival [22]. Copy number gain or amplification of 8q leads to increased expression of c-MYC which potentiates tumorigenesis. The 8q24 locus also contains single nucleotide polymorphisms that have an association with increased colon cancer risk [47].

Loss of 8p is commonly seen with gains of 8q/c-MYC with formation of an 8q isochromosome. The co-occurrence of these two abnormalities has a significantly elevated odds ratio of 3.9 to develop carcinomas [48]. 8p has been shown to encode multiple genes that inhibit tumorigenesis, such as DLC1, a tumor suppressor gene [27,28]. Loss of 8p has been demonstrated to change fatty acid and ceramide metabolism, which can allow for increased tumor growth and invasion [29]. 

Gains of 20q have also been demonstrated to play a major role in pathogenesis. Twenty percent of non-progressed adenomas had gains of 20q, while up to 60% of progressed adenomas and carcinomas had gains in 20q [23]. Due to gain of 20q, multiple genes mapped at 20q are overexpressed and are thought to play an important role in the transformation of adenomas to carcinomas: C20orf24, AURKA, TH1L, ADRM1, C20orf20, and TCFL5. C20orf24 and TCFL5 gene functions have not been well characterized [23]. However, AURKA overexpression has been shown to induce centrosome amplification and aneuploidy while TH1L helps regulate A-Raf kinase, which is involved in MEK/ERK pathway activation which leads to cell proliferation. ADRM1 encodes a cell adhesion molecule that helps comprise the 26S proteosome while C20orf20 binds to MRG15/MRGX proteins and is associated with cancer cell growth [24]. 

SRC is another oncogene expressed on chromosome 20q and encodes a non-receptor protein kinase leading to tumor progression and metastasis [25]. BCL2L1, also located on 20q, has a much higher copy number in colorectal cancers compared with adenomas, which translates to a statistically significant higher protein expression. This gene is thought to be involved in adenoma to carcinoma progression as a regulator of apoptosis [26]. 

18q21 loss is also an early event in colorectal cancer development, seen in up to 70% of primary colorectal cancer [6]. This region expresses the DCC and SMAD4 genes. DCC encodes for a nectrin-1 receptor and functions as a tumor suppressor gene with apoptotic ability, while SMAD4 regulates the TGF-β pathway to limit tumor growth and invasion [30].

### 3.2. Disease Progression

Various CNAs have been noted in the progression of colorectal cancer from early to advanced stages. Deletions or loss of 5p15.1, which correspond to FAM134B, a tumor suppressor gene, are noted in colorectal cancers and are significantly lower compared to nonneoplastic tissues or adenomas [33,34]. Moreover, deletions of 5p15.1 are found to be more common with more advanced T stage, N stage, and AJCC stages; this also translates to significantly decreased protein expression with advanced stage colorectal cancer, further suggesting this gene’s importance in disease progression [33].

FHIT, located on 3p14.2, controls apoptosis as a tumor suppressor, where deletions of FHIT appear more prevalent with advancing colorectal cancer stage. Stage III tumors have been found to have a higher deletion of FHIT (24.3%) compared to stage II tumors (3.3%), suggesting it plays an important role in disease progression [31]. Expression levels of FHIT are reduced in colorectal adenocarcinoma, suggesting an association with copy number deletion, where lower FHIT protein levels are seen with Dukes’ stages C and D and lymph node metastasis [49]. 

Loss of 4p also becomes more prominent in the progression of tumors from early Dukes’ stage to more advanced Dukes’ stage in one study [32]. Tumor suppressor genes are thought to be located on 4p; however, this is an area where further study is needed [50]. Losses on 4p may have an association with p53 mutations [51].

### 3.3. Distant Metastasis

While 8q gain and 8p loss are highly involved in initial pathogenesis, they appear to also play a role in disease progression in colorectal cancer. The 8q23–24 locus is noted to have significantly higher chromosomal gains specifically in patients with lymph node involvement compared to those without lymph node involvement (70% vs. 7%, respectively) [52]. One of the genes that may be amplified with 8q24 is PRL-3, which is expressed at higher levels in metastatic disease compared to localized disease [37]. PRL-3 is involved in glycolysis, improving glucose metabolism and lactate production, which further promotes tumor metastasis [38]. Copy number variation, specifically loss, of TNFRSF10C, located on 8p21, has been associated with lymph node involvement with an odds ratio of 18.8 (95% CI: 8.4–42.1) and distant metastasis with an odds ratio of 4.8 (95% CI: 2.1–10.8) [42]. This gene plays a role in inhibiting apoptosis through inhibition of intracellular signaling [43]. 

17p deletions are also involved in transition to advanced disease. Sixty percent of advanced colorectal cancer were found to have 17p deletions, significantly higher than 15% in early-stage colorectal cancer [44]. 17p loss is more prevalent in Dukes’ stage D and distant metastasis, where 93% of patients with 17p deletion had lymph node metastasis, significantly higher than 65% of patients without 17p deletion [45]. Patients with liver metastasis were also more likely to have 17p11.2 deletion compared to those with nonmetastatic disease (67% vs. 10%) [46]. P53 is located on 17p and is a well-known tumor suppressor gene, where loss or deletion of p53 is involved in colorectal cancer progression.

Amplification of WNK1, noted on chromosome 12p13.33, is also more frequent in cases of liver metastasis [32]. WNK1 is thought to facilitate MAP kinase signaling and cell cycle progression [32]. Deletion of 22q11.2 is more frequent in liver metastasis compared to nonmetastatic disease (22% vs. 0%) [46]. Increase in copy number between 11q13.3 and 11q22.3 is associated with increased nodal metastasis [39]. One study noticed that amplifications of ERBB2, FGFR1, PIK3CA, and CDK8 genes were present in metastatic sites, and not in the paired primary tumor, suggesting that these amplifications, which facilitate signaling in pathways including MAPK, PI3K/AKT, and Wnt/β-catenin, may also have a role in tumor metastasis [35,36,40]. 

A signature panel of three genes (S100PBP and CSMD2 from chromosome 1 and TGFBI from chromosome 5) is thought to promote metastasis, specifically to the liver. The loss of these three genes was significantly associated with synchronous liver metastasis and also predicted relapse free survival after hepatectomy in metastatic colorectal cancer [41]. S100PBP loss has been shown to lead to increased cancer invasion in vivo. CSMD2 has shown activity as a tumor suppressor and TGFBI has been shown to inhibit tumor cell invasion, where loss of these genes may lead to tumor progression [41]. Other CNAs associated with distant metastasis of colorectal cancer include loss of 14p and gain of 1q and 19 [32].

### 3.4. Microsatellite Stable Tumors and Consensus Molecular Subtypes (CMS)

Further investigation into CMS subtype has revealed some differences in CNAs between the groups, which further emphasize the distinct nature of these subtypes in microsatellite stable (MSS) tumors. CMS1 (“MSI Immune”) often has microsatellite instability as well as frequent BRAF mutations and CpG methylator phenotype and is thought to have the best prognosis. CMS2 (“Canonical”) has strong WNT/MYC pathway upregulation and high chromosomal instability. CMS3 (“Metabolic”) has frequent KRAS mutations and low chromosomal instability. CMS4 (“Mesenchymal”) is characterized by TGF-beta activation, stromal infiltration, and confers the worst prognosis [53]. 

CMS2 MSS has been shown to have high expression of CNAs, while CMS3 MSS tumors tend to have lower expression of CNAs [54,55,56]. CMS2 and CMS4 have been shown to have higher levels of CNAs than CMS1 and CMS3 for MSS disease [54]. Loss of 14q, 17p, 18p, and 18q and amplification of 5p and 20q are seen more frequently in CMS2 compared to CMS4 and gain of 10p and 13q amplifications are seen more frequently in CMS4 for MSS disease [56]. Often times, samples can be CMS unclassified, and these tend to have low CNA expression [55].

### 3.5. Mucinous and Microsatellite Instable Tumors

The changes that have been discussed are mostly in conjunction with microsatellite stable (MSS) adenocarcinoma. However, mucinous adenocarcinoma and microsatellite instable (MSI-H) colorectal adenocarcinomas appear to have different copy number profiles compared to MSS tumors. Mucinous cancers have been shown to have a reduced number of CNAs, 1.5 times lower than adenocarcinoma (*p* = 0.002). Additionally, mucinous tumors are less likely to have gain of chromosome 20q and loss of chromosome 18p [57]. 

MSI-H tumors tend to have a normal karyotype, compared to MSS tumors. MSS tumors are more likely to have deletions in 1p22, 4q26, and 15q21 with additional CNAs in 20p, 8p, and 18q [47]. However, there have been some similar chromosomal changes reported with the two tumor types, such as gains of 8q24, 16q24.3, and 20q13 and loss of 5q21. Although, gain of 22q13 has been seen more frequently in MSI-H tumors [23]. 

MSI-H tumors are distinct in their high mutation burden and the presence of mutation-associated neoantigens, which can provoke a T cell mediated immune response [58]. The CNA expression in MSI-H cancers reflects a corresponding upregulation in genes related to immune response as well as a downregulation in genes related to metabolism and cell–cell adhesion compared to MSS tumors [59]. These differences may help explain the activity of immunotherapy in MSI-H tumors.

### 3.6. CNA in Inflammatory Bowel Disease Associated CRC

It is known that patients with inflammatory bowel disease are at increased risk for developing colitis associated CRC, which appears to have a different CNA profile compared to sporadic CRC. For example, 5q22.2 loss, 17q loss, and Myc amplification are more common in colitis associated CRC [60,61]. These patients are also more likely to have near triploid or tetraploid karyotype [60]. However, there are some CNA characteristics that appear to be common in both colitis associated CRC and sporadic CRC, such as 8q amplification and 12p gain [62]. Interestingly, no significant difference in CNA events has been observed between those with Crohn’s vs. ulcerative colitis [60]. 

CNAs appear to be involved in the pathogenesis of colitis associated CRC. Microarray analysis has revealed a more than 13-fold difference in gains and more than 3-fold difference in losses of CNAs in patients with colitis associated CRC as well as an increase in total number of CNAs in colitis associated CRC compared to those considered to be low risk for developing colitis associated CRC [63]. As our understanding of CNAs in inflammatory bowel disease and CRC improves, hopefully we will be able to improve therapeutic options for the subset of patients with colitis associated CRC.

## 4. Prognostic Value of CNAs in Colorectal Cancer

### 4.1. Good Prognosis

Although 20q amplification has been shown to play a role in the pathogenesis of colorectal cancer, it is thought to be a good prognostic factor. Gain and amplification of chromosome 20q have both shown association with greater overall survival (OS) in patients with stage III or IV colorectal cancer [21]. Interestingly, 20q amplification is inversely associated with KRAS, NRAS, and BRAF mutations, which may explain a more favorable prognosis [30]. Although, a correlation between 20q amplification and the presence of p53 and APC mutations has been noted [25].

Higher levels of 20q amplification have been associated with longer OS in patients with stage IV MSS and left sided colon cancer, while gains in chromosome 20q11.21-q13.33 region are associated with improved OS in stage III disease [25,31]. Gain of ASXL1, also located on 20q11.21, thought to be a tumor suppressor, is associated with a favorable prognosis; patients with ASXL1 gain had a mean survival time of 48 months, significantly longer than 41 months in patients with ASXL1 negative colorectal cancer [64]. ASXL1 gain has also demonstrated a positive correlation with ASXL1 mRNA expression (R^2^ = 0.58) [64]. Presence or lack of 20q amplification has been shown to correlate with other mutations which may be of prognostic relevance: 72% of cases with amplification of 20q also had chromosome instability and p53 mutation, significantly higher than 44% in 20q nonamplified cases; 55% of cases without 20q amplification also had KRAS mutations, significantly higher than 29% in 20q amplified cases; 6.8% cases with 20q amplification had BRAF mutations, whereas none were noted in those without 20q amplification [65].

Gain of 7p11.2, reflective of EGFR, has also been associated with significantly longer progression free survival (PFS) to anti-EGFR therapy and OS [66,67]. Both 13q gain and 1p36 loss were shown to be associated with improved OS [68]. 

Gain of ERCC1, located on 19q13, is thought to be involved in enhanced nucleotide excision repair and increased sensitivity to platinum agents. It has been associated with significantly longer OS, interestingly only in patients with stage III colon cancer, but not in rectal cancer [69].

### 4.2. Poor Prognosis

Deletions on chromosome 10p15.3-p14 and 19p13.12, thought to correspond with tumor suppressor genes, were associated with decreased OS in stage II/III colorectal cancers [35,69,70]. Amplification of SKI, on chromosome 1p36, which represses TGF-β signaling, has shown an association with worse OS (HR 2.6, 95% CI: 1.2–5.6) and disease-free survival (DFS) (HR 2.1, 95% CI: 1.0–4.3) [71].

Loss of chromosome 4p or 4q has demonstrated associations with significantly shorter DFS in patients with colorectal cancer with an odds ratio of 2.1 (95% CI: 1.1–4.0) while also demonstrating increased propensity for lymph node metastasis in rectal cancer after chemoradiation [51,72].

Gains of c-MYC, corresponding with chromosome 8q24, also confers aggressive disease, where copy number gain ≥ 4.0 c-MYC copies/nucleus is associated with decreased OS (HR 1.8, 95% CI: 1.1–2.8) [22]. Deletion of 8p has also been shown to lead to decreased survival in sporadic colorectal cancer [73]. Specifically in rectal cancer, deletion of 8p is associated with significantly decreased metastasis free survival at 8 years (47.2% vs. 80.8%, HR 4.9, 95% CI: 1.5–15.8) and cancer specific survival at 8 years (53.1% vs. 85%, HR 3.5, 95% CI: 1.08–11.3). This chromosome deletion corresponded with significant decreased expression of 97 genes located on chromosome 8p [74]. Two of these genes, MTUS1 (8p22) and PPP2CB (8p12) are potential tumor suppressor genes [75].

The MET gene facilitates tumor invasion and metastasis in colorectal cancer, and gains of the MET gene, which corresponds with chromosome 7q31, also confer poor prognosis [76]. In patients who underwent curative surgical resection for colorectal cancer, a worse PFS (HR 2.0, 95% CI: 1.1–3.6) and OS (HR 2.2, 95% CI: 1.2–4.1) was seen with MET copy number gain ≥ 4 [77].

Deletion of 18q overall appears to be a poor prognostic marker in colorectal cancer [78]. However, various genes on chromosome 18 confer varying prognoses. SMAD7 is located on chromosome 18q, where deletion is associated with significantly better survival outcome and amplification is associated with significantly worse survival [79]. This gene is thought to inhibit cell growth arrest, allowing proliferation to occur [21]. Loss of CADH-7 on chromosome 18q also appears to be associated with favorable prognosis, with improved DFS (HR 0.4, 95% CI: 0.2–0.9) and OS (HR 0.3, 95% CI: 0.1–0.7) [79]. This suggests that CADH-7 may have an oncogenic role in colorectal cancer. However, loss of DNAM-1, which normally enhances T cell activity, leads to worse DFS (HR 2.0, 95% CI: 1.05–3.8) and OS (HR 2.4, 95% CI: 1.2–4.9) [78]. Moreover, deletion of BRUNOL4 at 18q12.2 and CD226, both on chromosome 18q, are associated with worse survival outcomes [21]. The prognostic role of these CNAs have been summarized in Table 2.

Higher copy numbers of mitochondrial DNA were also found to confer significantly poor prognosis in those with advanced stage colorectal cancer compared to those with low copy numbers (HR 2.5; 95% CI: 1.04–6.1). Higher mitochondrial DNA content also correlated with higher TNM stages (HR 3.0, 95% CI: 1.6–5.0) and liver metastasis (HR 2.1, 95% CI: 1.2–4.6) [81].

## 5. Predictive Value of CNAs in Colorectal Cancer

### 5.1. Favorable Response to Therapy Based on CNA

Amplification of ERBB2, or the HER2 gene, is seen in up to 5% of KRAS wild type colorectal cancer [82]. Recent trials have demonstrated promising results with HER2 directed therapy in ERBB2 amplified colorectal cancer. The HERACLES trial was a phase II trial that investigated the combination of trastuzumab and lapatinib in 27 heavily pretreated colorectal cancer patients with ERBB2 amplification [83]. A 30% objective response rate was noted, while 44% of patients achieved stable disease. Those with an ERBB2 copy number > 9.45 were noted to have much greater PFS (HR 0.67, 95% CI: 0.6–0.8) [83]. The MyPathway trial, a phase IIA multiple basket study, also showed encouraging results with targeting ERBB2 amplification in heavily pretreated colorectal cancer patients: the combination of trastuzumab and pertuzumab was found to have a response rate of 37.5% [84]. More recently, antibody–drug conjugate, trastuzumab deruxtecan in DESTINY-CRC01 trial showed excellent activity in patients with HER2-positive metastatic colorectal cancer [85]. 

Gain or amplification of PIK3CA, located on 3q26, appears to be a favorable characteristic in colorectal cancer. Patients with gain/amplification of PIK3CA are noted to have significantly greater OS; for patients treated with adjuvant chemotherapy or radiation, those with PIK3CA gain or amplification were noted to have significantly greater survival compared to those who did not have gain/amplification [86]. Moreover, PIK3CA gain and amplification did correlate with PIK3CA protein expression and there was no correlation with PIK3CA mutation [86]. 

EGFR copy number gain ≥4.0/nucleus confers significantly improved PFS with anti-EGFR therapy (HR 0.2, 95% CI: 0.1–0.5) in colorectal cancer patients refractory to chemotherapy [87]. Disease control has been seen in 73% of patients with high EGFR copy number treated with anti-EGFR therapy, compared to 20% of patients with low EGFR copy number [88]. Another study has shown that when treated with panitumumab, a mean EGFR copy number gain <2.5/nucleus led to a significantly shorter PFS and OS [89]. 

Conversely, KRAS copy number has implications in KRAS wild type patients treated with cetuximab. Those with copy number gains have demonstrated worse survival, while those with copy number losses have exhibited a good response with cetuximab [90]. In colorectal cancer cell lines, KRAS copy number gains have been associated with an 11-fold increase in RAS-GTPase activity; similarly, KRAS codon 12 or 13 mutations lead to a 12-fold increase in RAS-GTPase activity [90].

Loss of 18p11.32–18q11.2 and 18q12.1–23 were both associated with significantly increased response to first line chemotherapy with capecitabine and irinotecan [68]. Low expression of TYMS, a gene located on 18p11.32, is also associated with improved response to fluorouracil-based therapy in metastatic colorectal cancer [68]. Loss of 1p36 was also more frequent in responders (31%) vs. non-responders (6%) [68]. 

Variations of SMAD4 copy number also has implications of predicting response to 5-FU therapy in colorectal cancer. SMAD4, as mentioned previously, is located at 18q21 and may be a tumor suppressor in colorectal cancer. Patients with SMAD4 deletion were found to have significantly longer DFS (HR 2.9, 95% CI: 1.02–8.1) with adjuvant 5-FU [79].

For patients treated with chemotherapy and bevacizumab, loss of 18q12.1–18q21.32 was associated with significantly longer PFS [91]. In two separate cohorts receiving bevacizumab, loss of 18q11.2–q12.1 was associated with increased PFS (cohort 1: HR 0.54, *p* = 0.01; cohort 2: HR 0.55, *p* = 0.02) [91]. Deletion of 18q has shown correlation with increased vascularization of tumors, which may explain why bevacizumab, as an anti-angiogenesis agent, leads to improved responses in patients with loss of 18q [92]. Loss of DNAM-1, also located on 18q, has also been shown to lead to lower risk of death with adjuvant 5-FU therapy (HR 0.5, 95% CI: 0.3–1.0) [78].

### 5.2. Poor Response to Therapy Based on CNA

MET and ERBB2 amplification have been seen in patients with resistance to anti-EGFR therapy, also suggestive of their predictive value [93,94]. ERBB2 amplification leads to persistent ERK1/2 signaling which leads to anti-EGFR resistance. However, concurrent treatment with anti-HER2 therapy and anti-EGFR therapy, may overcome this resistance: pertuzumab and lapatinib has demonstrated meaningful activity in patient-derived xenografts of colorectal cancer resistant to cetuximab [95,96]. MET encodes a tyrosine kinase receptor for hepatocyte growth factor to facilitate tumorigenesis. Addition of MET inhibitors in cases of MET amplification has also been shown to overcome anti-EGFR resistance in patient-derived xenografts [97]. 

In proximal colon cancer with depressed morphology, significant enrichment of CNAs in c-MYC, CCNA1, and BIRC7 has been observed. C-MYC, as discussed previously, is a major oncogenic driver; CCNA1, located on chromosome 13q13.1, is also involved in tumor invasion and metastatic spread, while BIRC7, located on chromosome 20q13.1, plays a role in cancer cell proliferation, invasion, and metastasis. When treated with oxaliplatin in the first line setting, patients with these CNAs had significantly shorter PFS and OS [98]. 

Amplification of STRAP, located on 12p12.3, has been observed to lead to worse survival in stage II/III colorectal cancer patients being treated with adjuvant fluorouracil-based chemotherapy [71]. STRAP stands for serine threonine receptor-associated protein and functions as a TGF-β pathway inhibitor. STRAP synergizes with SMAD7, which is thought to sustain colon cancer cell growth and survival [99]. Conversely, patients with diploidy or deletion of STRAP were found to have a trend towards lower risk of death (HR 0.4, 95% CI: 0.2–1.0) [99]. 

PTEN is a tumor suppressor, located on 10q23, that controls cell proliferation in the PI3K/AKT pathway [100]. When comparing loss of PTEN vs. no loss, no difference was observed in PFS or OS [101]. For patients receiving irinotecan as second line therapy, increase in copy number of TOP1, located on chromosome 20q12, was not found to have a statistically significant difference in objective response [102]. 

**Figure 2:**
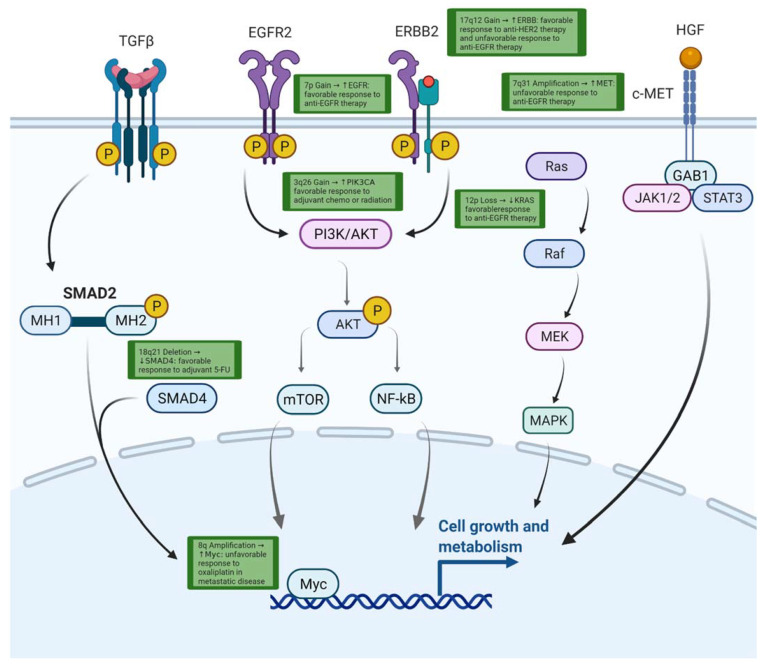
Pathway of molecular pathways affected by CNAs and associated response to therapies. Created with BioRender.com.

CNA burden may also have predictive value regarding response to immunotherapy in colorectal cancer. One study identified that those with low CNA burden (CNA ≤ 10) had significantly improved PFS to immunotherapy compared to those with high CNA burden (CNA > 10). Further study revealed that the group with low CNAs had a different immune environment with higher IFN-γ with upregulation of genes involved in lymphocyte regulation and checkpoint pathways [103]. The predictive role of these CNAs has been summarized in Table 3 and relevant pathways are illustrated in Figure 2.

## 6. Conclusions

In summary, CNAs appear to play a significant role in colorectal adenocarcinoma. Various CNAs are involved in the pathogenesis of colorectal cancer initiation and progression. Additionally, differences in CNAs appear to confer varying prognoses and have also demonstrated predictive value with certain therapeutic agents. Often times, CNAs appear to correlate with levels of mRNA and protein expression. An improved understanding of the multi-faceted roles of CNAs in colorectal cancer can hopefully lead to a better understanding of the disease as well as the development of therapeutic options that improve patient outcome.

## Figures and Tables

**Table 1:** Copy number alterations with associated genes and functions in the pathogenesis of colorectal cancer.

Pathogenesis Stage	Copy Number Alterations	Associated Gene	Associated Protein [21]	Signal Pathway (If Known)	Biologic Activity and Function (If Known)
Adenoma to Carcinoma	Gain of 8q [22]	*c-MYC*	c-MYC		Transcription factor that facilitates cell proliferation and survival
	Gain of 20q [23,24,25,26]	*ADRM1*	Adrm1		Component of 26S proteasome that encodes a cell adhesion molecule
		*AURKA*	Aurora Kinase A	Wnt and Ras-MAPK	Serine threonine kinase that induces centrosome amplification and aneuploidy
		*BCL2L1*	Bcl-2-like protein 1		Caspase activation inhibitor that regulates apoptosis
		*C20orf20*	MRG/MORF4L-binding protein		Histone acetylation that binds to MRG15/MRGX proteins and is associated with cancer cell growth
		*C20orf24*	Respirasome Complex Assembly Factor 1		Mitochondrial respirasome assembly
		*SRC*	Src	STAT3, PI3K, RAS	Non-receptor protein kinase that promotes tumor progression and metastasis
		*TCFL5*	Transcription factor-like 5 protein		Transcription factor
		*TH1L*	Negative elongation factor C/D	MEK/ERK	Helps regulate A-raf kinase, which is involved in *MEK/ERK* pathway activation
	Loss of 8p [27,28,29]	*DLC1*	Rho GTPase-activating protein 7	MAPK	GTPase-activating protein involved in fatty acid and ceramide metabolism
	Loss of 17p [22]	*p53*	p53	p53	Tumor suppressor, transcription factor
	Loss of 18q21 [6,30]	*DCC*	DCC		Nectrin-1 receptor that facilitates apoptosis
		*SMAD4*	SMAD4	*TGF-β*	Transcription factor and tumor suppressor that regulates *TGF-β* pathway
Disease Progression	Loss of 3p14.2 [31]	*FHIT*	Bis(5’-adenosyl)-triphosphatase	PI3K/AKT	Dinucleoside triphosphate hydrolase that regulates apoptosis
	Loss of 4p [32]				
	Deletion of 5p15.1 [33,34]	*FAM134B*	Reticulophagy regulator 1	AKT	Autophagy receptor and tumor suppressor
Distant Metastasis	Gain of 1q [32]				
	Amplification of 3q26 [35]	*PIK3CA*	PIK3CA	PI3K/AKT	Kinase involved in *PI3K/AKT* signaling pathway
	Amplification of 8p11.23 [36]	*FGFR1*	FGFR1	MAPK, PI3K/AKT	Receptor tyrosine kinase for fibroblast growth factors
	Gain of 8q [37,38]	*PRL-3*	PRL-3	NF-kB	Protein tyrosine phosphatase that facilitates glycolysis, glucose metabolism, and lactate production
	Amplification of 11q [39]				
	Amplification of 12p13.33 [32]	*WNK1*	WNK1	WNK	Serine/threonine protein kinase that facilitates cell cycle progression
	Amplification of 13q12 [35,40]	*CDK8*	CDK8	mTOR	Protein phosphorylator that regulates β-catenin activity
	Amplification of 17q12 [35]	*ERBB2*	ERBB2	PI3K/AKT	Receptor tyrosine kinase that leads to cellular growth
	Gain of 19 [32]				
	Loss of 1p35 [41]	*S100PBP*	S100PBP		Interacts with S100 calcium-binding protein P
		*CSMD2*	CSMD2		Tumor suppressor involved in complement cascade
	Loss of 5q31 [41]	*TGFBI*	TGFBI	PI3K/AKT	Precursor of TGFBI and inhibits tumor cell invasion
	Loss of 8p21 [42,43]	*TNFRSF10C*	TNFRSF10C		Receptor for the cytotoxic ligand TRAIL and inhibits apoptosis
	Loss of 14p [32]				
	Deletion of 17p [44,45,46]	*p53*	p53	p53	Tumor suppressor
	Deletion of 22q11.2 [46]				

**Table 2:** Copy number alterations and their associated genes and functions with prognostic roles in colorectal cancer.

Prognosis	Copy Number Alteration	Gene	Associated Protein [21]	Signaling Pathway	Biological Activity and Function (If Known)
Favorable Prognosis	Gain of 7p11.2 [66,67]	*EGFR*	EGFR	EGFR	Receptor tyrosine kinase binding ligand that facilitates tumor progression
	Gain of 19q13 [69]	*ERCC1*	ERCC1		Enhanced nucleotide excision repair protein
	Gain/amplification of 20q [25,31,64]	*ASXL1*	ASXL1		Chromatin regulator and tumor suppressor
	Loss of 18q [79]	*CADH-7*	Cadherin-7		Cell–cell adhesion protein
Poor Prognosis	Amplification of 1p36 [71]	*SKI*	Ski	*TGF-β*	Represses *TGF-β* signaling
	Gain of 7q31 [76,77]	*MET*	Hepatocyte growth factor receptor	HGF/MET	Receptor tyrosine kinase that facilitates tumor invasion and metastasis
	Gain of 8q24 [22]	*c-MYC*	c-MYC		Transcription factor that facilitates cell proliferation and survival
	Amplification of 18q [21,79]	*SMAD7*	SMAD7	*TGF-β*	Represses *TGF-β* signaling and inhibits cell growth arrest
	Loss of 4p/4q [51,72]				
	Deletion of 8p22 [75]	*MTUS1*	MTUS1	ERK	Potential tumor suppressor
	Deletion of 8p12 [75]	*PPP2CB*	PPP2CB	ERK	Serine threonine protein phosphatase as a potential tumor suppressor
	Deletion of 10p15.3-p14 [31,80]				
	Loss of 18q [78]	*DNAM-1*	CD226 antigen		Cell surface receptor for Nectin 2 that enhances T-cell activity
	Deletion of 18q12.2 [21]	*BRUNOL4*	BRUNOL4		
	Deletion of 19p13.12 [70]				

**Table 3:** Copy number alterations and their associated genes and functions with predictive roles in colorectal cancer.

Response Prediction	Copy Number Alteration	Gene	Associated Protein [21]	Signaling Pathway	Therapy
Predictive of Favorable Response	Gain of 3q26 [85]	*PIK3CA*	PIK3CA	PI3K/AKT	Adjuvant chemotherapy or radiotherapy in early-stage colorectal cancer
	Gain of 7p11.2 [66,67]	*EGFR*	EGFR	EGFR	Anti-*EGFR* therapy in metastatic colorectal cancer
	Gain of 17q12 [83,84]	*ERBB2*	ERBB2	PI3K/AKT	Anti-*HER2* therapy in metastatic colorectal cancer
	Loss of 12p12.1 [89]	*KRAS*	KRAS	RAS/RAF	Anti-*EGFR* therapy in metastatic colorectal cancer
	Loss of 18p11.32 [68]	*TYMS*	Thymidylate synthase		Fluorouracil-based therapy in metastatic colorectal cancer
	Loss of 18q [78]	*DNAM-1*	CD226 antigen		Adjuvant 5-FU in early-stage colorectal cancer
	Loss of 18q12.1–18q21.32 [90]				Chemotherapy plus bevacizumab in metastatic colorectal cancer
	Deletion of 18q21 [79]	*SMAD4*	SMAD4	*TGF-β*	Adjuvant 5-FU in early-stage colorectal cancer
Predictive of Poor Response	Amplification of 7q31 [92]	*MET*	Hepatocyte growth factor receptor	HGF/MET	Anti-*EGFR* therapy in metastatic colorectal cancer
	Amplification of 8q [22]	*MYC*	myc		Oxaliplatin in metastatic colorectal cancer
	Gain of 12p12.1 [89]	*KRAS*	KRAS	RAS/RAF	Anti-*EGFR* therapy in metastatic colorectal cancer
	Amplification of 12p12.3 [71]	*STRAP*	STRAP	*TGF-β*	Adjuvant fluorouracil-based chemotherapy in stage II/III colorectal cancer
	Amplification of 13q13.3 [97]	*CCNA1*	CCNA1		Oxaliplatin in metastatic colorectal cancer
	Amplification of 17q12 [94]	*ERBB2*	ERBB2	PI3K/AKT	Anti-EGFR therapy in metastatic colorectal cancer
	Amplification of 20q13.3 [97]	*BIRC7*	BIRC7		Oxaliplatin in metastatic colorectal cancer
